# Supplementary material for: Predictors of survival among breast cancer patients in Ethiopia: a systematic review and meta-analysis
Source: Front Oncol. 2025 Apr 3;14:1459613. doi: 10.3389/fonc.2024.1459613 (PMC12003360; doi:10.3389/fonc.2024.1459613)
Supplement: Supplementary file 4 [file Table2.docx]

**Supplementary S 3 file: Searching strategy**

| Google scholar | | | | Result |
| --- | --- | --- | --- | --- |
| Concept | | | Search detail |  |
| Concept1 map | Delayed presentation breast cancer patient | Keyword | " Delayed presentation breast cancer patient  "[tw] OR "late presentation of breast cancer patient"[ tw] OR "breast cancer care"[tw] OR “late stage diagnosis of breast cancer ”[tw] OR “timing of diagnosis breast cancer”[tw] OR “breast cancer”[tw] | 12043 |
|  |  | Mesh term | “delayed breast cancer presentation ”[Mesh] | 351 |
| Concept 3 map | Associated factors | Keyword | "Associated factors" [tw] OR "determinants"[tw] OR "Predictors"[tw] OR "Factors contributing"[tw] OR "correlates"[tw] | 4702 |
| Concept 3 map | Breastcancer patient | Keyword | "breast cancer caret"[tw] “Breastcancer patient”[tw] | 451 |
|  |  | Mesh term | "breast cancer patient"[MeSH Terms] | 780 |
| Concept 4 map | East Africa | Keyword | East Africa [tw] | 541 |
|  |  | Mesh term | "East Africa "[MeSH Terms] |  |
| Concept 5 map | Prevalence | Keywords | Prevalence[tw] or Magnitude[tw] OR Proportion[tw] | 5623 |
|  |  | Mesh term | "Prevalence"[Mesh] |  |
| (("we used Terms like "breast cancer," "associated factors," "predictors," "determinants," "contributing factors," "prevalence," "magnitude," "proportion," "delayed patient presentation," "late presentation breast cancer," "late diagnosis breast cancer," "late diagnosis of patient," "East Africa," In addition, eastern African countries, namely, Ethiopia, Ertriea, Sudan, South Sudan, Djibouti, Kenya, Rwanda, Zimbabwe, Tanzania, Uganda, Somalia, Burundi, Namibia, Botswana, Reunion, Mayotte, Seychelles,Madagaskar, Marituis and Democratic republic of Congo were also included to ensure a comprehensive search. We experimented and improved utilizing several test searches, combining related search phrases with Boolean operators like OR and combining distinct notions using Boolean operator AND. | | | | 2134 |
| PubMed | | | |  |
| Delayed presentation of breast cancer patient in East Africa. | | | | 342 |
| **Other database sources** | | | | 104 |
